# Case of *bla_KPC-12_*-carrying hypervirulent *Klebsiella pneumoniae* from bloodstream infection in China

**DOI:** 10.1093/jacamr/dlaf048

**Published:** 2025-04-05

**Authors:** Aoxiao Chen, Hanyu Wang, Jing Zhang, Yi Sun, Gongxiang Chen, Hongwei Zhou

**Affiliations:** Clinical Microbiology Laboratory, The Second Affiliated Hospital of Zhejiang University School of Medicine, Zhejiang University, Hangzhou, China; Beijing Key Laboratory of Detection Technology for Animal-Derived Food Safety, College of Veterinary Medicine, China Agricultural University, Beijing, China; Clinical Microbiology Laboratory, The Second Affiliated Hospital of Zhejiang University School of Medicine, Zhejiang University, Hangzhou, China; Clinical Microbiology Laboratory, The Second Affiliated Hospital of Zhejiang University School of Medicine, Zhejiang University, Hangzhou, China; Clinical Microbiology Laboratory, The Second Affiliated Hospital of Zhejiang University School of Medicine, Zhejiang University, Hangzhou, China; Clinical Microbiology Laboratory, The Second Affiliated Hospital of Zhejiang University School of Medicine, Zhejiang University, Hangzhou, China

Sir,

The ST11 clone is the most prevalent lineage among hypervirulent *Klebsiella pneumoniae* (hvKP) and carbapenem-resistant *K. pneumoniae* (CRKP) worldwide,^1^ with *bla_KPC-2_* playing a pivotal role in the development of CR-hvKP and hv-CRKP.^2^ According to the Comprehensive Antibiotic Resistance Database, 242 *bla_KPC_* variants have been identified globally, with *bla_KPC-2_* and *bla_KPC-3_* being the most widespread.^3^  *bla_KPC-12_*, a rare variant of *bla_KPC-2_*, is characterized by an L169M substitution in the Ω-loop, which may influence the resistance to ceftazidime/avibactam.^4^

Between 2017 and 2024, the NCBI database reported 22 *K. pneumoniae* strains carrying *bla_KPC-12_*, 21 of which were from China and 1 from Greece. Given the rarity of reports on *bla_KPC-12_* and the lack of documented fatal cases, we present a detailed analysis of a fatal infection caused by a hvKP strain harbouring *bla_KPC-12_*, focusing on its genetic context, resistance genes and virulence factors.

In January 2024, a *K. pneumoniae* strain (KP2414) was isolated from the blood culture of a 67-year-old female patient who had undergone heart valve surgery. The patient had been hospitalized in the intensive care unit for 3 months due to elevated postoperative inflammatory markers and several high-risk infection factors. During hospitalization, sputum cultures repeatedly tested positive for CRKP. The patient ultimately died from septic shock, despite treatment with the following combination of antibiotics sequentially: ceftazidime/avibactam, cefoperazone/sulbactam and ceftazidime/avibactam.

Antimicrobial susceptibility test revealed that KP2414 was susceptible only to ceftazidime/avibactam (MIC = 4/4 mg/L), tigecycline (1 mg/L) and polymyxin (≤0.5 mg/L), as detailed in Table S1 (available as Supplementary data at *JAC-AMR* Online). One possible explanation for its susceptibility to ceftazidime/avibactam is that the isolate has not yet undergone the induction required for resistance, as resistance to ceftazidime/avibactam in KPC-12 has been shown to develop after multiple passages under drug pressure.^4^

Genome sequencing analysis of KP2414 (Supplementary method) revealed that it belongs to the ST11, K19 type. It carries several virulence genes such as *iucA*, *rmpA* and *rmpA2*, as well as multiple resistance genes, including *fosA6* (fosfomycin resistance), *qnrS1* (quinolone resistance), *tet*(A) (tigecycline resistance), *aadA2* and *rmtB* (aminoglycoside resistance) and carbapenem resistance genes including *bla_KPC-12_*. Compared with the reference strain ATCC13883, KP2414 showed no differences in *OmpK35*. However, a glycine and aspartic acid insertion were found in the extracellular Loop 3 region of *OmpK36*, which likely increases the resistance to carbapenem antibiotics.

After four to five generations of passaging, KP2414 regained sensitivity to meropenem, most likely due to the increased fitness cost associated with the insertion of an amino acid in the L3 loop of *OmpK36*.^5^

The genetic environment of *bla_KPC-12_* in the Tn6929 transposon on the plasmid was analysed, as shown in Figure 1(b). Tn6929 is the most commonly reported transposon carrying *bla_KPC_* in China.^6^ The genetic environment of KP2414 consists of ▵Tn1755-5′-repB-orf-klcA-orf-korC-ISKpn6-*bla_KPC_*_-12_-▵Tn6376(ISKpn27). Insertions of 3-bp inverted repeat sequences were found in the flanking regions of ▵ISKpn27 and ▵Tn1755-5′, and six TGGACC insertion repeat sequences were identified at both the 5′ and 3′ ends of the entire genetic environment. Upstream of *bla_KPC-12_*, G and C nucleotide promoter-binding sites were identified at 39 and 250 bp, respectively. The −35/−10 elements of P1 and P2 promoter regions (TAATCC/TTACAT and TTGAC/AATAAT) is consistent with the transcriptional environment of *bla_KPC-2_*, as previously reported by Wang *et al*.^7^

**Figure 1.**
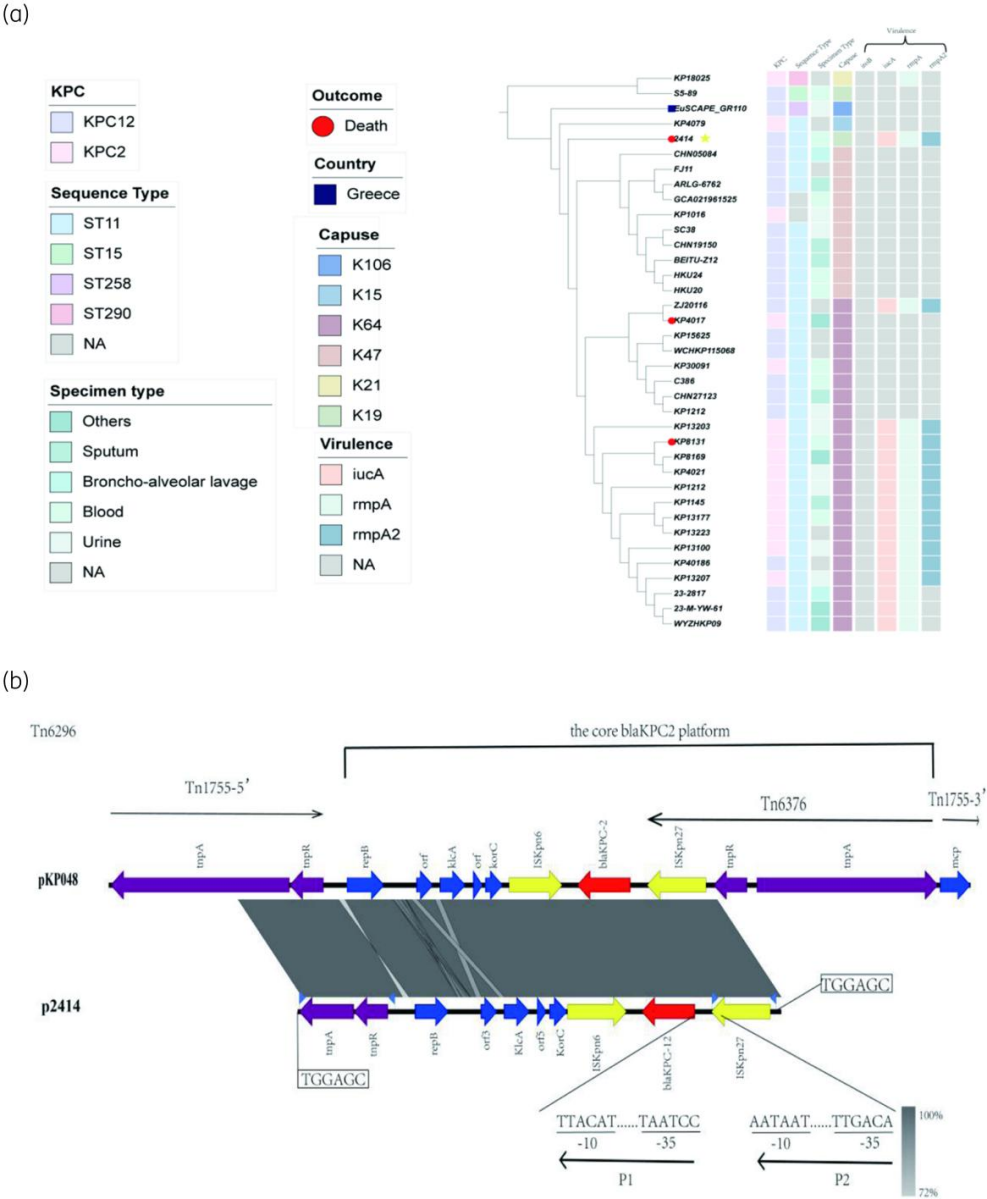
(a) Phylogenetic tree based on core genome SNPs of 38 *K. pneumoniae* clinical isolates carrying *bla_KPC-2_* and *bla_KPC-12_*. Blue squares represent isolates from Greece, while all other isolates are from China. Red circles indicate cases with fatal outcomes, while outcomes for other isolates remain unspecified. (b) Genetic environments comparison of KP2414 and KP048 (carrying *bla_KPC-2_*). Regions with >99.0% homology are shaded in grey. Red arrows indicate *bla_KPC-2_* and *bla_KPC-12_*; blue arrows represent genes with unknown or other functions; purple arrows indicate genes encoding transposase functions (e.g. tnpA and tnpR); yellow arrows represent insertion sequences, with triangles marking inverted repeat sequences.

Phylogenetic analysis included 15 ceftazidime/avibactam-resistant *K. pneumoniae* strains carrying *bla_KPC-2_*,^8^ 22 strains of *K. pneumoniae* carrying *bla_KPC-12_* from the NCBI database and KP2414 (Figure 1a). Among the 17 hvKP strains analysed, six were found to carry *bla_KPC-12_*. Notably, *bla_KPC-12_* was detected in both the patient and hospital wastewater, underscoring the potential risk of full-chain transmission. hvKP is known for its propensity to cause bloodstream infections, and its co-occurrence with *bla_KPC-12_* poses severe challenges for clinical treatment. To the best of our knowledge, KP2414 represents the first reported case of *bla_KPC-12_*-carrying hvKP from bloodstream infection in China. Therefore, while *bla_KPC-2_* and *bla_KPC-3_* remain globally prevalent, the complex transposon-mediated resistance structure of *bla_KPC-12_* and its emerging prevalence in China, coupled with its full-chain transmission potential, represent significant threats to both public health and clinical management.

## Supplementary Material

dlaf048_Supplementary_Data

## Data Availability

The genome assemblies of KP2414 have been deposited in the NCBI and are registered under BioProject accession NO. PRJNA1203937. All data are available from the corresponding authors upon reasonable request.

## Supplementary data

Table S1 is available as Supplementary data at *JAC-AMR* Online.
