# Supplementary material for: Case of blaKPC-12-carrying hypervirulent Klebsiella pneumoniae from bloodstream infection in China
Source: JAC Antimicrob Resist. 2025 Apr 5;7(2):dlaf048. doi: 10.1093/jacamr/dlaf048 (PMC11971478; doi:10.1093/jacamr/dlaf048)
Supplement: dlaf048_Supplementary_Data [file dlaf048_supplementary_data.zip › Supplementary_method_CLEAN.docx]

**Supplementary material**

**Methods**

**Strain isolation**

A sterile 10 µL inoculation loop was used to pick a loop of blood culture broth and inoculate it onto a China Blue selective agar plate containing 0.3 µg/mL meropenem, followed by overnight incubation at 35°C. MALDI-TOF-MS (Bruker Daltonics, Germany) identification confirmed a single colony of *Klebsiella pneumoniae*. Virulence genes *iucA*, *rmpA*,*rmpA2* and *iroB* were identified via PCR.

| Primer | Primer (5’- 3’) | Gene | Product size (bp) | Source |
| --- | --- | --- | --- | --- |
| rmpA-F | ACTCGCCTACCTCTGCTTCA | *rmpA* | 516 | [1] |
| rmpA-R | CTTGCATGAGCCATCTTTCA |  |  |  |
| rmpA2-F | GTGCAATAAGGATGTTACATTA | *rmpA2* | 430 | [2] |
| rmpA2-R | GGATGCCCTCCTCCTG |  |  |  |
| iucA-F | AATCAATGGCTATTCCCGCTG | *iucA* | 239 | [2] |
| iucA-R | CGCTTCACTTCTTTCACTGACAGG |  |  |  |
| iroB-F | ATCTCATCATCTACCCTCCGCTC | *iroB* | 235 | [2] |
| iroB-R | GGTTCGCCGTCGTTTTCAA |  |  |  |

[1] Candan ED, Aksöz N. Klebsiella pneumoniae: characteristics of carbapenem resistance and virulence factors. *Acta Biochim Pol*. 2015;**62**(4):867-74.

[2] Russo TA, Olson R, Fang CT et al. Identification of Biomarkers for Differentiation of Hypervirulent Klebsiella pneumoniae from Classical K. pneumoniae. *J Clin Microbiol*. 2018;**56**(9).

**Antimicrobial susceptibility testing**

The antimicrobial susceptibility testing was performed using the broth microdilution method, with interpretation standards based on the Clinical and Laboratory Standards Institute (CLSI) guidelines. The quality control strain used was Escherichia coli ATCC 25922.

**Whole genome sequencing & Statistical analysis**

Genomic DNA was extracted using the Magen bacterial DNA extraction kit (HiPure Bacterial DNA Kit), and Illumina NovaSeq PE150 was used for next-generation sequencing. The genome was assembled de novo using SPAdes v3.15.1,^1^ and annotation was performed using the Rapid Annotation using Subsystem Technology (RAST) database (<https://rast.nmpdr.org/>).^2^ The ST type, capsular serotype, and virulence genes of the strain were analyzed using Kleborate v3.^3^ Antimicrobial resistance (AMR) genes were identified using ABRicate v1.0.0. ^4^ A phylogenetic tree was constructed based on core genome alignment with Snippy v4.6.0, and the core genome single nucleotide polymorphisms (cgSNPs) were calculated using Gingr and snp-dists software.^5, 6^ The core genome was extracted using Snippy by aligning the genomes to a reference. SNPs were then called from the core genome alignment. A phylogenetic tree was constructed from the resulting SNP matrix using RAxML with the GTR+G model and 1000 bootstrap replicates. The phylogenetic tree was visualized and beautified using iTOLv7 (<https://itol.embl.de/>).^7^ The gene environment surrounding *bla_KPC-12_* was compared using EasyFig v2.2.5.^8^

1. Bankevich A, Nurk S, Antipov D et al. SPAdes: a new genome assembly algorithm and its applications to single-cell sequencing. *J Comput Biol*. 2012;**19**(5):455-77.

2. Overbeek R, Olson R, Pusch GD et al. The SEED and the Rapid Annotation of microbial genomes using Subsystems Technology (RAST). *Nucleic Acids Res*. 2014;**42**(Database issue):D206-14.

3. Lam MMC, Wick RR, Watts SC et al. A genomic surveillance framework and genotyping tool for Klebsiella pneumoniae and its related species complex. *Nat Commun*. 2021;**12**(1):4188.

4. Zankari E, Hasman H, Cosentino S et al. Identification of acquired antimicrobial resistance genes. *J Antimicrob Chemother*. 2012;**67**(11):2640-4.

5. Feng Y, Zou S, Chen H et al. BacWGSTdb 2.0: a one-stop repository for bacterial whole-genome sequence typing and source tracking. *Nucleic Acids Res*. 2021;**49**(D1):D644-d50.

6. Treangen TJ, Ondov BD, Koren S et al. The Harvest suite for rapid core-genome alignment and visualization of thousands of intraspecific microbial genomes. *Genome Biol*. 2014;**15**(11):524.

7. Letunic I, Bork P. Interactive Tree of Life (iTOL) v6: recent updates to the phylogenetic tree display and annotation tool. *Nucleic Acids Res*. 2024;**52**(W1):W78-w82.

8. Sullivan MJ, Petty NK, Beatson SA. Easyfig: a genome comparison visualizer. *Bioinformatics*. 2011;**27**(7):1009-10.
