# Supplementary material for: Case of blaKPC-12-carrying hypervirulent Klebsiella pneumoniae from bloodstream infection in China
Source: JAC Antimicrob Resist. 2025 Apr 5;7(2):dlaf048. doi: 10.1093/jacamr/dlaf048 (PMC11971478; doi:10.1093/jacamr/dlaf048)
Supplement: dlaf048_Supplementary_Data [file dlaf048_supplementary_data.zip › Supplementary_Table_CLEAN.docx]

**Case of *bla_KPC-12_*-Carrying Hypervirulent *Klebsiella pneumoniae* from Bloodstream Infection in China**

**Supplementary Material**

**Table 1. MIC results for KP2414.**

|  | KP2414 | |
| --- | --- | --- |
| Antibiotics | MIC | Interpretation |
| IPM | 8 | R |
| MEM | 64 | R |
| ETP | 128 | R |
| CMZ | ≥128 | R |
| CAZ | ≥128 | R |
| CTX | ≥128 | R |
| TZP | ≥256/4 | R |
| SCF | ≥256/128 | R |
| CAV | 4/4 | S |
| FEP | ≥64 | R |
| PB | ≤0.5 | S |
| TGC | 1 | S |
| CIP | ≥32 | R |
| AK | ≥128 | R |
| ATM | ≥128 | R |

* MIC, minimal inhibitory concentration, units are mg/L. “R”, “S” representes resistant and sensitive, respectively. IPM, imipenem; MEM, meropenem; ETP, ertapenem; CMZ, cefmetazole; CAZ, ceftazidime; CTX, cefotaxime; TZP, piperacillin/tazobactam; SCF, cefoperazone/sulbactam; CAV, ceftazidime/avibactam; FEP, cefpirome; PB, polymyxin; TGC,tigecycline; CIP, ciprofloxacin; AK,amikacin; ATM, aztreonam
